# Sulfated Polysaccharides in the Freshwater Green Macroalga *Cladophora surera* Not Linked to Salinity Adaptation

**DOI:** 10.3389/fpls.2017.01927

**Published:** 2017-11-13

**Authors:** Paula X. Arata, Josefina Alberghina, Viviana Confalonieri, María I. Errea, José M. Estevez, Marina Ciancia

**Affiliations:** ^1^Universidad de Buenos Aires, Facultad de Agronomía, Departamento de Biología Aplicada y Alimentos, Cátedra de Química de Biomoléculas, Buenos Aires, Argentina; ^2^Universidad de Buenos Aires, Facultad de Ciencias Exactas y Naturales, Departamento de Ecología, Genética y Evolución, Instituto IEGEBA (UBA-CONICET), Buenos Aires, Argentina; ^3^Instituto Tecnológico de Buenos Aires, Departamento de Ingeniería Química, Buenos Aires, Argentina; ^4^Fundación Instituto Leloir-IIBBA-CONICET, Buenos Aires, Argentina; ^5^CONICET-Universidad de Buenos Aires, Centro de Investigación de Hidratos de Carbono (CIHIDECAR), Buenos Aires, Argentina

**Keywords:** green alga, *Cladophora*, cell walls, sulfated polysaccharides, freshwater environment

## Abstract

The presence of sulfated polysaccharides in *cell walls* of seaweeds is considered to be a consequence of the physiological adaptation to the high salinity of the marine environment. Recently, it was found that sulfated polysaccharides were present in certain freshwater *Cladophora* species and some vascular plants. *Cladophora* (Ulvophyceae, Chlorophyta) is one of the largest genera of green algae that are able to grow in both, seas and freshwater courses. Previous studies carried out on the water-soluble polysaccharides of the marine species *C. falklandica* established the presence of sulfated xylogalactoarabinans constituted by a backbone of 4-linked β-L-arabinopyranose units partially sulfated mainly on C3 and also on C2 with partial glycosylation, mostly on C2, with terminal β-D-xylopyranose or β-D-galactofuranose units. Besides, minor amounts of 3-, 6- and/or 3,6-linked β-D-galactan structures, with galactose in the pyranosic form were detected. In this work, the main water soluble cell wall polysaccharides from the freshwater alga *Cladophora surera* were characterized. It was found that this green alga biosynthesizes sulfated polysaccharides, with a structure similar to those found in marine species of this genus. Calibration of molecular clock with fossil data suggests that colonization of freshwater environments occurred during the Miocene by its ancestor. Therefore, the presence of sulfated polysaccharides in the freshwater green macroalga *C. surera* could be, in this case, an adaptation to transient desiccation and changes in ionic strength. Retention of sulfated polysaccharides at the cell walls may represent a snapshot of an evolutionary event, and, thus constitutes an excellent model for further studies on the mechanisms of sulfation on cell wall polysaccharides and environmental stress co-evolution.

## Introduction

It has been proposed that the presence of sulfated polysaccharides in cell walls of seaweeds and marine angiosperms, absent in terrestrial plants, is a consequence of the physiological adaptation to the marine environment (Kloareg and Quatrano, 1988; Aquino et al., 2005) due to a strong environmental pressure. Most of sulfated polysaccharides present in seaweed (e.g., carrageenans) are well known to exhibit the solubility characteristics typical of hydrophilic colloids due to the presence of hydroxyl and sulfate groups in their backbones. Under certain conditions, they can form hydrogels, three-dimensional networks capable of maintaining a large amount of water. This moisture retention capacity is believed to be important for macroalgal desiccation resistance. Besides, their solubility characteristics are greatly affected by the salt form of their sulfate groups. Moreover, they show an important tendency to retain Ca^2+^and Mg^2+^, in agreement with their known capacity to retain these salts (Kloareg and Quatrano, 1988; Estevez et al., 2004). Sulfated polysaccharides produced by marine angiosperm *Ruppia maritima* Loisel disappeared when the plant was cultivated in the absence of salt (Aquino et al., 2011). On the other hand, the glycophyte *Oryza sativa* Linnaeus, when exposed to salt stress (200 mM NaCl) did not induce the biosynthesis of sulfated polysaccharides, but increased the concentration of carboxylated polysaccharides of the pectin type (Aquino et al., 2011). These data suggested that the presence of sulfated polysaccharides in marine plants is an adaptation to high-salinity environments, which may have been conserved during plant evolution from marine green algae (Aquino et al., 2011).

In an opposite way, very recently, it was found that, at least the green alga *Cladophora glomerata* and also *Ulva flexuosa* (Ulvophyceae, Chlorophyta), from two different freshwater environments (Nan river in Thailand and Lake Oporzynskie in Poland), with no detectable salt, were able to synthesize sulfated polysaccharides (Pankiewicz et al., 2016; Surayot et al., 2016). In both investigations, although the presence of sulfated polysaccharides was well established, the fine structures of polysaccharides where these sulfate groups were detected, were not conclusive, and further studies are required to confirm these findings. In addition, in the vascular plant *Eichhornia crassipes*, a native plant from Amazonas, also known as water hyacinth, collected in a freshwater tropical environment with no salinity, high quantities of sulfated polysaccharides were found in petioles, rhizome, and roots (Dantas-Santos et al., 2012). These highlighted examples suggest possible roles for cell wall sulfated polysaccharides in organisms exposed to stressful conditions different to salinity stress, like long periods of desiccation, high temperature exposure, transient changes in ionic strength in the water media (caused by water evaporation in small microsites), and so on.

The branched genus *Cladophora* (Ulvophyceae, Chlorophyta) is one of the largest genera of green algae that are able to grow in both, marine and fresh-water environments (Boedeker et al., 2016). We then decided to test if one species of *Cladophora* that grows in freshwater environments still retained the ability to modify its polysaccharides by the addition of sulfate groups, independently of the salinity stress. We chose to characterize the main cell wall components of *C. surera* E.R. Parodi and E.J. Cáceres due to the abundance of this freshwater alga in the southern part of Buenos Aires Province (Argentina) for at least 30 years (Parodi and Cáceres, 1991, 1995). Previous studies carried out on the water-soluble polysaccharides of several species of *Cladophora* from marine environments established the presence of sulfated xylogalactoarabinans (Percival and McDowell, 1981; Sri Ramana and Venkata Rao, 1991; Arata et al., 2016). Recently, the structure of the polysaccharides from *C. falklandica* was studied in detail, and it was found that they are constituted by a backbone of 4-linked β-L-arabinopyranose units partially sulfated mainly on C3 and also on C2 with partial glycosylation mostly on C2 with single β-D-xylopyranose, single β-D-galactofuranose units, or short β-D-galactofuranose chains comprising (1→5)- and/or (1→6)-linkages. Besides, minor amounts of 3-, 6- and 3,6-linked β-D-galactan structures, with galactose in the pyranosic form were detected.

Here, we have found that, although *C. surera* grows in a freshwater environment with no detected salt (as NaCl), it still biosynthesizes highly sulfated cell wall polymers. These results open new questions about the roles of sulfated polysaccharides, not necessarily linked to salt–stress, but possibly associated to a response to desiccation stress and changes in ionic strength of the environment in fluctuating freshwater habitats.

## Materials and Methods

### Algal Sample

Specimens of *C. surera* Parodi *et* Cáceres subsp. nov. were collected in Las Cascadas, Necochea, Buenos Aires Province (38°27′39′′ S 58°45′39′′ W) in April 2015 (**Supplementary Figure S1**). The water salinity at this site was < 1‰, measured with a Salinity Refractometer S/MIII, Cat No. 2441, ATAGO CO., LTD. Sulfate content of water at the collection site was determined by ion exchange chromatography with conductimetric detection using a DIONEX DX-100 chromatography system (Sunnyvale, CA, United States) with an AS4A column (4mm × 250 mm), an AMMS-II micromembrane suppressor; elution was carried out with 1.8 mM Na_2_CO_3_/1.7 mM NaHCO_3_, at a flow rate of 2 mL min^-1^.

*Cladophora surera* grew free floating in the freshwater course. Sporophytic and gametophytic plants are isomorphic. Measurements of length and width of cells from the principal axis and branches were within the range reported for this species by Parodi and Cáceres (1995). Gametangia developed from the upper cells in ultimate ramifications with similar dimensions and appearance to the vegetative cells. The samples used in this work were in the vegetative state. Thalli of the algae were washed with distilled water and analyzed for epiphytic and epizoic contaminants in a Nikon AFX-II macroscope (Nikon, Japan).

### Phylogenetic Position of *Cladophora surera*

**FIGURE 1:**
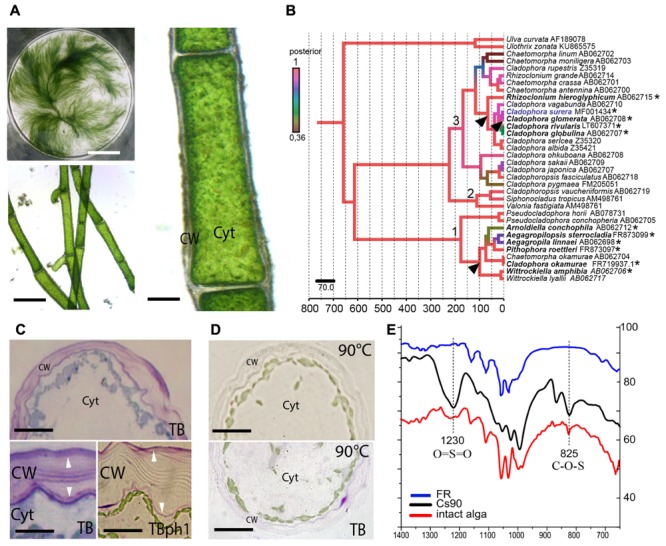
Cell wall sulfated polysaccharides in the freshwater alga *Cladophora surera*. **(A)** General aspect of the alga. Scale bar = 5 cm. Branched filaments (Scale bar = 150 μm) and cell morphology with a well-developed cell wall (CW). Cyt = cytoplasm. Scale bar = 20 μm. **(B)** Bayesian tree, showing the phylogenetic position of *C. surera* collected in Quequén Grande river (Buenos Aires Province, Argentina). 1: *Aegagropila* clade; 2: *Siphonocladus* clade; 3: *Cladophora* clade. Scale-time bar underneath indicates million years. Left color bar indicates higher (red) to lower (green) posterior probabilities. ^∗^Facultative or permanent fresh-water species. Arrowhead: putative events of freshwater colonization. Accession numbers are indicated with species names. **(C)** *In situ* distribution of sulfated polysaccharides, stained with TB*O* at pH7 (for anionic polysaccharides) and pH1 (for sulfated polysaccharides). Arrowheads indicate the presence of sulfated polysaccharides in two CW layers. Cyt = cytoplasm. Scale bar = 15 μm (top picture). Scale bar = 5 μm (bottom pictures). **(D)** Cross-sections of a *C. surera* cell after hot-water extraction (at 90°C) without staining (top panel) and stained with TB*O* at pH1 (bottom panel). Scale bar = 15 μm. **(E)** Attenuated Total Reflection-Fourier Transformed Infra-Red (ATR-FTIR) spectra of intact alga, of the hot-water CW extract (Cs90), and of the residue after exhaustive aqueous extraction (FR). Diagnostic bands for the presence of sulfate ester groups are highlighted at 1230 cm^-1^, due to asymmetric stretching of O = S = O and at 825 cm^-1^, due to stretching of C_ecuatorial_-O-S and/or C_primary_-O-S.

Genomic DNA was obtained from fresh algal material of *C. surera* using REDExtracts-N-Amp Tissue PCR Kit (SIGMA-ALDRICH). The nuclear-encoded small subunit (LSU) rDNA gene fragment was amplified and sequenced using primers and temperature profile as indicated in Boedeker and Immers (2009). Sequences were inspected and aligned using Geneious version 7.0 (Biomatters). SSU sequence was deposited in Gene Bank under accession number MF001434. The position of *C. surera* was investigated through the phylogenetic analyses of Thirty-two species of Cladophorales (Ulvophyceae), representing marine, brackish and freshwater environments (Accession numbers in **Figure 1B**). Two species from Ulvophyceae (*U. curvata* and *Ulothrix zonata*) were included as outgroups. Phylogenetic analyses of molecular characters were performed through Bayesian analysis (BA) as implemented in BEAST v1.6.2 (Drummond and Rambaut, 2007; Heled and Drummond, 2010). The model of sequence evolution was GTR+I+G (Rodríguez et al., 1990; Yang, 1994; Gu et al., 1995). Calibration of molecular clock was performed using the estimated minimum age of 600–570 million years for Cladophorales (van den Hoek and Chihara, 2000; Leliaert et al., 2007) based on fossil data. An uncorrelated relaxed clock model and a log-normal fossil calibration was used during tree searches. The number of Markov Chain Monte Carlo (MCMC) iterations was 30000000, from which the first 3000000 were discarded as non-converged burn-in; nodal support values were given as posterior probabilities.

### Light Microscopy and Histochemistry

For light microscopy (LM) semithin sections (10 μm) were mounted on glass slides and then observed with a Carl Zeiss Axiolab microscope (Carl Zeiss, Jena, Germany). The staining procedures used in LM histochemical characterization based on Krishnamurthy (1999), were carried out on the fixed tissues described above and included Toluidine Blue *O* (TB*O*; 0.05% w/v) in 0.1 m HCl at pH 1.0 that stains sulfated polysaccharides (red–purple, c metachromasia), and TB*O* at pH7 (for negatively charged polysaccharides). Hot water extraction at 90°C was carried out on cross-sections, which were then stained in conditions described above.

### Extraction and Purification of the Polysaccharides

Algal samples of *C. surera* were dried in open air. The milled algae (100 g) were extracted twice with EtOH 70% (20g/L) for 3 h at room temperature. The residue from the alcohol extraction was extracted for 3 h with H_2_O (20 g/L) at 90°C, giving extract Cs90 (20.1 g). Cs90 was dissolved in water (4.5 mg/ml). A residue (Ri) was separated from the supernatant, which was chromatographed on DEAE-Sephadex A-25. The supernatant was applied to a column (90 cm × 1.5 cm id), previously stabilized in H_2_O. The first elution solvent was water and then NaCl solutions of increasing concentration up to 4 M. Fractions of 4 ml were collected. Finally, the phase was boiled in 4 M NaCl solution. The presence of carbohydrates in the samples was detected by the phenol sulfuric acid method (Dubois et al., 1956); after obtaining blank readings, the eluant was replaced by another with higher concentration of NaCl. Seven fractions (F1-F7) were obtained, dialyzed (molecular weight cut off 3,500) and freeze dried (Supplementary Figure S2).

### Chemical Characterization

The total sugars content was analyzed by the phenol-sulfuric acid method (Dubois et al., 1956). Sulfate was determined turbidimetrically (Dodgson and Price, 1962). Alternatively, ion exchange chromatography with conductimetric detection was used: the sample was hydrolyzed in 2 M CF_3_COOH at 121°C for 2 h, evaporated to dryness under nitrogen and redissolved in high purity water from a Milli-Q system. A DIONEX DX-100 chromatography system (Sunnyvale, CA, United States) was used with an AS4A column (4 × 250 mm), an AMMS-II micromembrane suppressor and a conductivity detector, elution was carried out with 1.8 mM Na_2_CO_3_/1.7 mM NaHCO_3_, at a flow rate of 2 mL min^-1^. The absence of pyruvic acid and uronic acids was confirmed using the colorimetric determinations of Koepsell and Sharpe (1952) and Filisetti-Cozzi and Carpita (1991). The protein content was measured by microanalysis to determine the amount of nitrogen, a factor of 5 was applied to calculate the amount of protein, according to Angell et al. (2016). The configuration of galactose and arabinose was determined by the method of Cases et al. (1995) through their diastereomeric acetylated 1-deoxy-1-(2-hydroxypropylamino) alditols. To determine the monosaccharide composition, samples were derivatized to the alditol acetates (Stevenson and Furneaux, 1991).

### Methylation Analysis

The sample (10–20 mg) was converted into the corresponding triethylammonium salt (Stevenson and Furneaux, 1991) and methylated according to Ciucanu and Kerek (1984). The sample was dissolved in dimethylsulfoxide; finely powdered NaOH was used as base. The methylated samples were submitted to reductive hydrolysis and acetylation to give the alditol acetates in the same way as the parent polysaccharides (Stevenson and Furneaux, 1991).

### Gas Chromatography

GC of the alditol acetates were carried out on a Agilent 7890A gas-liquid chromatograph (Avondale, PA, United States) equipped with a flame ionization detector and fitted with a fused silica column (0.25mm i.d. × 30 m) WCOT-coated with a 0.20 mm film of SP-2330 (Supelco, Bellefonte, PA, United States). Chromatography was performed: from 200 to 240°C at 2°C min^-1^, followed by a 10-min hold for alditol acetates. For the partially methylated alditol acetates, the initial temperature was 160°C, which was increased at 1°C min^-1^ to 210°C and then at 2°C min^-1^ to 230°C. N_2_ was used as the carrier gas at a flow rate of 1 mL min^-1^ and the split ratio was 80:1. The injector and detector temperature was 250°C.

### Gas Chromatography-Mass Spectrometry

GC–MS of the partially methylated alditol acetates was performed on a Agilent 7890A gas-liquid chromatograph equipped the SP-2330 interfaced to a Agilent 5977A Series mass spectrometer, working at 70 eV. The flow rate was 1.3 ml min^-1^, the injector temperature was 250°C. Mass spectra were recorded over a mass range of 30–500 amu.

### Desulfation of Cs90

The reaction was carried out by the microwave-assisted method described by Navarro et al. (2007). The sample (40 mg) was converted to the pyridinium salt and dissolved in 10 ml of DMSO containing 2% of pyridine. The mixture was heated for 10 s intervals and cooled to 50°C (× 6). It was dyalized 3 days against tap water and then 24 h against distilled water (MWCO 3,500) and lyofilized. An aliquot was methylated as described above without previous isolation of the product.

### Partial Acid Hydrolysis of Ri

The reaction was carried out according to Bilan et al. (2007). The sample (100 mg) was heated in 1% CH_3_COOH (20 mL) for 4 h at 100°C, the solution was neutralized with NaHCO_3_, dialyzed, and lyophilized to give RiH (78.2 mg). RiH was fractionated by anion exchange chromatography on DEAE-Sephadex A-25 in a similar way as F1, in this case, five fractions were obtained (RiH-F1-RiH-F5).

### ATR-FTIR Spectroscopy

Samples were analyzed on a Thermo Scientific Nicolet 6700 spectrometer equipped with a smart ARK (Attenuated Reflectance Kit) accessory using a standard ZnSe crystal and a DTGS KBr detector. The spectra were recorded in the 650–4000 cm^-1^ range, and the spectral resolution was 4 cm^-1^. Data were processed by using the software Origin Pro 9.0.0.

### NMR Spectroscopy

500 MHz^1^H NMR, proton decoupled 125 MHz ^13^C NMR spectra, and two-dimensional NMR experiments (HSQC, HMBC, and COZY) were recorded on a Bruker AM500 at room temperature, with external reference of TMS. The samples (20 mg) were exchanged in 99.9% D2O (0.5 mL) four times. Chemical shifts were referenced to internal acetone (δ_H_ 2.175, δ_CH3_ 31.1). Parameters for ^13^C NMR spectra were as follows: pulse angle 51.4°, acquisition time 0.56 s, relaxation delay 0.6 s, spectral width 29.4 kHz, and scans 25,000. For ^1^H NMR spectra the parameters were: pulse angle 76°, acquisition time 3 s, relaxation delay 3 s, spectral width 6250 Hz and scans 32. 2D spectra were obtained using standard Bruker software.

## Results and Discussion

### *Cladophora surera* Biosynthesizes Sulfated Polysaccharides

Specimens of the green alga were collected in a freshwater environment (Quequén Grande river, 38°27′39′′ S 58°45′39′′ W) located in Buenos Aires Province, Argentina (**Supplementary Figure S1**). Based on the morphological characters, it was assigned to *C. surera* (**Figure 1A**; Parodi and Cáceres, 1991, 1995).

In order to gain further insight on the taxonomical identity, a molecular characterization of the SSU (nuclear-encoded small subunit rDNA) gene fragment of the collected species was carried out and its phylogenetic position within the Cladophorales was investigated. The Bayesian tree obtained (**Figure 1B**) recovered with high Posterior Probabilities (PP) the three major groups previously described by Boedeker et al. (2012): the “*Aegagropila* clade,” the “*Siphonocladus* clade” and the “*Cladophora* clade.” *C. surera* is resolved within the latter group, and particularly within a lineage of four species that can be mainly found in freshwater/brackish environments (Hanyuda et al., 2002; Boedeker et al., 2012). Within the order Cladophorales, the boundary between marine and freshwater environments was crossed at least two to three times (van den Hoek, 1963; Logares et al., 2007; Hayakawa et al., 2012; Boedeker et al., 2016; **Figure 1B**). Calibration of molecular clock with fossil data suggests that colonization of freshwater environments probably occurred 11.4 million years ago (MYA; 4–25 MYA 95% PP) by the ancestor of the four freshwater species closely related to *C. surera* (**Figure 1B**); or even before, at about 65.5 MYA (35–115 MYA 95%PP), during the diversification of the more basal species *Rhizoclonium hieroglyphicum*.

*Cladophora surera* showed a thick cell wall with two fibrillar-like layers delimiting a middle amorphous region. TB*O* staining suggested the presence of sulfated polysaccharides concentrated in both marginal cell wall layers (**Figure 1C**). Hot water extraction (at 90°C) carried out on cross-sections greatly suppressed TB*O* staining (**Figure 1D**). In agreement, the hot water extract (Cs90) obtained from *C. surera* that represents 20.1% (w/w) of the algal dry weight (Supplementary Figure S2) contained high amounts of sulfate (17%, as SO_3_Na, which correspond to ∼4% w/w of the algal dry weight), in accordance with the histochemical stainings.

Finally, Attenuated Total Reflection-Fourier Transformed Infra-Red (ATR-FTIR) spectrum of the dry milled alga, as well as that of extract Cs90 (**Figure 1E**) showed the bands diagnostic for the presence of sulfate ester groups at 1230 cm^-1^, which was assigned to asymmetric stretching of O = S = O, and at 825 cm^-1^, due to stretching of C_ecuatorial_-O-S and/or C_primary_-O-S (Prado Fernández et al., 2003). In agreement, after exhaustive sequential extraction with water (at 90°C) and with alkaline solutions, both IR bands (825 and 1230 cm^-1^) mostly disappeared in the remaining cell wall residue (FR) confirming that sulfated polysaccharides are confined in the aqueous extracts (**Figure 1E**).

All these results together confirmed the presence of high quantities of sulfated polysaccharides in cell walls of freshwater macroalga *C. surera*. The water salinity at the collection site was < 1‰ (w/w; as NaCl) and the sulfate content of the water was ∼1 mM, while usually in marine waters there are high sulfate concentrations (∼25–28 mM) (Bochenek et al., 2013). These results suggested that sulfated polysaccharides in some *Cladophora* species (at least in *C. glomerata*, and now in *C. surera*) could be synthetized in response to other stress factors, like desiccation and changes in ionic strength of the environment, not linked to high salinity levels as a stable environmental condition.

### Sulfated Polysaccharides from *Cladophora surera* Have Common Structural Features with those of Marine Species of the Genus *Cladophora*

In order to characterize in depth the structure of these sulfated polysaccharides, with emphasis in the position of the sulfate groups on the carbohydrates backbone, the monosaccharide composition of the main cell wall extract obtained with hot water (Cs90) was determined. Cs90 is composed by arabinose and galactose as major monosaccharides, also important amounts of xylose were present (**Table 1**). Methylation analysis of Cs90 showed major quantities of 2,3-di-*O*-methyl-, 2-*O*-methyl-, 3-*O*-methyl-, and non-methylated arabinose, which correspond to 4-linked non-substituted, 3-substituted, 2-substituted, and disubstituted arabinose units (17, 28, 20, and 28 per 100 arabinose units, respectively) in the polysaccharide backbone. Desulfation of Cs90 gave Cs90D (**Table 1**), which by methylation analysis showed an important increase in 4-linked non-substituted arabinose units (A, ∼50% of the total arabinose units), and a decrease of 3-substituted (A3S) and 2,3-disubstituted units (A2,3S), while the percentage of 4-linked 2-substituted (A2X + A2R, R = chains up to four residues of Galp) units also increased to 38%. These results indicate that sulfate groups were mainly linked to C3 of the arabinopyranose units and in minor amounts, to C2 and C3, while some arabinose units were sulfated on C3 and substituted with single units of xylose or galactose on C2. Only minor amounts of galactofuranose units were detected. These units were previously found in important quantities in the arabinan of *C. falklandica* (Arata et al., 2016), and they were also detected in *C. rupestris* (Percival and McDowell, 1981, and references therein). Besides, methylation and desulfation–methylation analysis suggested the presence of short side chains of 3- and 3,6-linked galactopyranose units, partially sulfated on C4.

**Table 1:** Yields and analyses of Cs90, the hot water extract from *Cladophora surera*, its desulfated derivative (Cs90D), and FiH3 a modified cell wall fraction.

Sample	NaCl, M	Yield^2^%	Carbohydrates%	Sulfate (as SO_3_Na) %	Monosaccharide composition Moles %				
					Rha	Ara	Xyl	Gal	Glc
Cs90^1^	–	20.9	31.3	17.3	5	43	17	27	7
Cs90D		42.1	51.9	4.0	3	57	14	18	7
FiH3	2.0	14.8	45.7	32.2	7	66	12	15	–


*^1^Protein content: 14.4%.^2^Yield of Cs90 is given for 100 g of dry algal material, of Cs90D, per 100 g of Cs90, and of FiH3, for 100 g of RiH (see **Table 2**)*.

**FIGURE 2:**
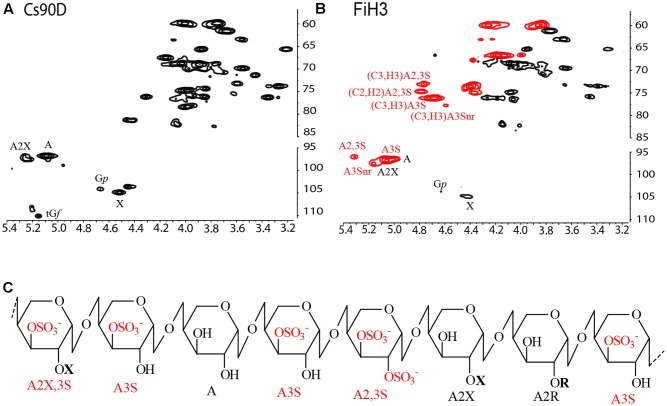
Average structure of sulfated xylogalactoarabinans, characterized by linkage analysis and Nuclear Magnetic Resonance (NMR) spectroscopy. ^1^H-^13^C Heteronuclear Single Quantum Coherence (HSQC) NMR spectra of **(A)** Cs90D and **(B)** FiH3 CW fractions. **(C)** Proposed structure of sulfated xylogalactoarabinan isolated from *C. surera.* Sulfated arabinose units are highlighted in red. Structural units: 4-linked β-L-arabinopyranose (A), 4-linked β-L-arabinopyranose sulfated at C-3 (A3S), 4-linked β-L-arabinopyranose disulfated at C2 and C3 (A2,3S), 4-linked β-L-arabinopyranose substituted with single units of β-D-xylopyranose at C2 (A2X), 4-linked β-L-arabinopyranose disubstituted with β-D-xylopyranose at C2 and sulfate at C3 (A2X3S), 4-linked β-L-arabinopyranose substituted at C2 with short chains up to four residues of β-L-galactopyranose (G*p*; A2R) at C2. Non-reducing end A3S residues (nr) are also shown in the spectrum of FiH3.

As Cs90 had a limited solubility in water, it was not possible to carry out NMR spectroscopic analysis of this extract, however, good spectra were obtained from Cs90D (**Figure 2A** and **Table 2**), which allowed to determine that all the major monosaccharide units described above were in the β-configuration, and to confirm the linkages between them.

**Table 2:** Signal assignment of the HSQC NMR spectra of Cs90D and FiH3.

Structural unit	Chemical shifts (ppm)					
	C-l/H-1	C-2/H-2	C-3/H-3	C-4/H-4	C-5/H-5,5′	C-6/H-6,6′
**CS90D^1^**						
4-linked β-L-Ara*p* (A)	97.3/5.03	69.1/3.89	68.9/4.02	75.2/4.02	60.4/4.01,3.81	
4-linked β-L-Ara*p* with β-D-Xyl*p* on C2 (A2X)	98.4/5.18	78.3/4.02	67.9/4.14	75.2/4.02	60.4/4.01,3.81	
T-β-D-Xyl*p* (X)	105.7/4.45	74.1/3.33	76.3/3.39	70.2/3.58	66.0/3.88,3.24	
T-β-D-Gal*f*(G*f*)	108.8/4.98	82.0/4.04	77.7/3.97	84.5/3.96	71.6/3.75	63.7/3.58,3,65
5-linked β-D-Gal/(5G)	108.9/5.21			82.9/4.14	76.2/3.89	62.0/3.70
3-linked β-D-Gal*p* (3G)	104.4/4.41	71.1/3.72	83.0/3.80	69.4/4.18	75.8/3.63	62.0/3.70
3,6-linked β-D-Gal*p* (3,6G)	104.9/4.60	71.1/3.72	83.0/3.80	69.4/4.18	74.7/3.85	70.4/3.98, 3.86
TP-D-Galp *(T-Gp)*	103.6/4.51	71.7/3.47	73.6/3.61		75.8/3.65	62.0/3.70
**FiH3^2,3,4^**						
4-linked β-L-Ara*p* (A)	97.3/5.03	69.1/3.89	68.9/4.02	75.4/4.02	60.4/4.03,3.85	
4-linked β-L-Ara*p* with β-D-Xyl*p* on C2 (A2X)	98.4/5.18	78.3/4.02	67.9/4.14	75.4/4.02	60.4/4.03,3.85	
T-β-D-Xyl*p* (X)	105.7/4.45	74.1/3.33	76.3/3.39	70.2/3.58	66.0/3.88,3.24	
4-linked β-L-Ara*p* 3-sulfate (A3S)	97.6/5.09	67.4/4.12	76.7/4.63	74.2/4.34	60.9/3.78,4.20	
4-linked β-L-Ara*p* 2_,_3-disulfate (A2,3S)	96.8/5.34	75.2/4.73	73.6/4.71			
Tn-β-L-Ara*p* 3-sulfate (A3S-nr)	98.6/5.19	67.3/3.94	78.2/4.54	68.4/4.32	63.8/4.25,4,17	
T-β-D-Gal*f* (T-G*f*)	109.6/5.14	82.4/4.08	76.9/3.93	82.6/3.95	71.2/3.72	63.7/3.60,3,65


*^1^Ratio Ara:Ara2X in Cs90D **∼**1.0:0.5, calculated from the ^1^H NMR spectrum. ^2^Ratio Ara2.3S:Ara2X+TAra3Snr:Ara3S:Ara in Fi-H3 ∼1.0:1.2:6.0:1.5 calculated from the ^1^H NMR spectrum. ^3^Peaks corresponding to sulfated carbons are highlighted in red. ^4^A signal at δ 81.0/4.28 was attributed to C3/H3 of 6-linked β-D-galactopyranose 3-sulfate units*.

Besides, a strategy comprising controlled acid hydrolysis, fractionation by anion exchange chromatography (Supplementary Figure S2), and structural elucidation of the fractions by methylation analysis and NMR spectroscopy (**Figures 2A,B** and **Table 2**) was performed. Similar structural features were found in all the fractions obtained, but in different quantities. The highly purified sample FiH3 is shown as example (**Figure 2B**, and **Tables 1, 2**), and confirmed the structural features of these polysaccharides. As expected for FiH3, a fraction which eluted from an anion exchange chromatographic column with 2 M NaCl (Supplementary Figure S2), it is highly sulfated, and disulfated arabinose units are present in important amounts. In addition, it was found that in this fraction the β-D-galactopyranose units were mostly 6-linked and sulfated on C3. This structure was proposed based on methylation analysis, which showed the presence of 2,4-di-*O*-methylgalactose, as major partially methylated galactose derivative, and by the absence of a signal at δ 83.0/3.80 which would correspond to 3-linked β-D-galactopyranose units. Moreover, a small signal at δ 81.0/4.28 is a strong evidence for the presence of the mentioned 6-linked 3-sulfated galactose units. This substitution pattern of the galactan moiety was similar to that found in marine *C. socialis* (Sri Ramana and Venkata Rao, 1991). On the other hand, in *C. falklandica*, most of the galactose was in the furanosic form, and only minor quantities of 3-linked mostly 6-sulfated galactopyranose units were detected. Here, the important amount of terminal galactopyranose units, detected by methylation analysis and confirmed in the NMR spectra of Cs90D (**Figure 2A** and **Table 2**), indicates possible small side chains of up to ∼4 units of the arabinan backbone. These results suggest that the most important structural differences between the sulfated polysaccharides from species of *Cladophora* studied so far are restricted to the galactose units, while the pyranosic arabinan backbone and its sulfation pattern is conserved.

In conclusion, sulfated xylogalactoarabinans from cell walls of the freshwater alga *C. surera* have structural features similar to those reported for the marine species of this genus. Based on the structural determination carried out by linkage analysis and NMR spectroscopy, it is proposed that *C. surera* biosynthesizes sulfated xylogalactoarabinans as shown in **Figure 2C**.

### Evolutionary Implications of the Presence of Sulfated Polysaccharides in *C. surera*

The presence of sulfated polysaccharides in *C. surera* is in agreement with two recent studies on *C. glomerata* from different freshwater environments (Nan river in Thailand and Lake Oporzynskie in Poland) (Pankiewicz et al., 2016; Surayot et al., 2016). In all three cases, they developed in freshwater environments. Sulfated polysaccharides are widespread in the cell walls of marine angiosperms, in marine algae (green, red, and brown seaweeds), in the extracellular matrix of vertebrate tissues, and in invertebrate species (Aquino et al., 2005; Ghandi and Mancera, 2008; Pomin and Mourao, 2008; Usov and Bilan, 2009; Ciancia et al., 2010; Usov, 2011). In the green alga *Lamprothamnium papulosum* (Characeae, Charophyta), which grows in water of fluctuating salinity, the extracellular sulfated mucilage increases in sulfate content with increasing salinity (Shepherd and Beilby, 1999; Shepherd et al., 1999), while in brown algae the concentrations of sulfate groups on fucans positively correlate with increasing exposure to the atmosphere in the intertidal zone, suggesting a role in desiccation resistance (Mabeau and Kloareg, 1987). *Ectocarpus subulatus* (Phaeophyta) isolated from a true freshwater environment (West and Kraft, 1996) undergoes major morphological, transcriptomic and metabolic changes under variable salinities, including alteration of the expression of genes encoding enzymes potentially involved in the sulfation or de-sulfation of cell wall polysaccharides (Dittami et al., 2012). It is now widely accepted that the occurrence of sulfated polysaccharides in phylogenetically distant taxa is a case of convergent adaptation to a broad range of environmental conditions, such as stable high ionic media (e.g., seawater for marine plants and algae) or transient changes in salt concentration linked to desiccation and high temperature stresses (e.g., in some cases of freshwater macroalgae and vascular plants) (Mabeau and Kloareg, 1987; Shepherd and Beilby, 1999; Shepherd et al., 1999; Aquino et al., 2011; Dantas-Santos et al., 2012; Pankiewicz et al., 2016; Surayot et al., 2016). From a physiological point of view, sulfated cell wall polysaccharides from marine plants and marine algae may confer an adaptive advantage through possible structural and osmotic functions that are linked to an environmental pressure. Terrestrialization, and the subsequent taxonomic proliferation of land plants, was predated by a divergence between the Chlorophyte (a group of green algae) and the Streptophyte lineages (which includes land plants). This is strongly correlated with a change in habitat preference from saline to freshwater conditions with low or no salt, as well as exposure to transient changes in ionic strength (e.g., caused by water evaporation in small microsites). These transitions are accompanied by a major alteration in cell wall polysaccharides composition such that, although the majority of marine Chlorophytes contain sulfated cell wall polysaccharides, they are largely absent from most of the freshwater Charophycean green algae and their descendants. The latter green algae contain large amounts of negatively charged pectins with carboxylated sugars (uronic acids) in their cell walls that could functionally replace some of the roles of sulfated polysaccharides (Domozych et al., 2012). Further studies to determine if sulfated polysaccharides are present in other freshwater algae are required to obtain a more comprehensive picture about their possible functions in the cell wall.

## Conclusion

Although further evidence is required, early emerging freshwater and terrestrial organisms may have had a demand for sulfur that was in excess in the marine environment, but not in terrestrial and freshwater habitats (Raven, 1997). Such requirement for sulfate may have become a limiting factor and, therefore, may have been selected in organisms in a terrestrial environment, potentially resulting in the lack of carbohydrate sulfotransferases and sulfatases from the genomes of freshwater algae and land plants and sulfated polysaccharides from their cell walls (Popper et al., 2011). The case of both freshwater *Cladophora* species may indicate that not always the presence of sulfated polysaccharides provides an ionic barrier to high levels of salts, but it could be acting in response to other environmental factors like desiccation and transient changes in ionic strength media. It is important to note, that the effect of transient desiccation could give an important temporary increase in the ionic strength of the medium. Also, the high temperatures, in which plants in tropical regions develop, could cause desiccation. So, all these factors could be linked to high ionic strength environments, permanent or lasting only a short period of time. Hence, retention of sulfation capacity in freshwater *Cladophora* species could be due to this factor, as well as to water retention capacity. Colonization of freshwater environments by the ancestor of the four freshwater *Cladophora* species closely related to *C. surera* probably occurred during the Miocene. Retention of sulfated polysaccharides at the cell walls of freshwater green macroalgae may represent a more general phenomenon, which constitutes an excellent model for further studies on the mechanisms of sulfation on cell wall polysaccharides and stress factors in freshwater environments co-evolution. Further genomic and physiological studies are required to understand the molecular basis of the retention of sulfated polysaccharides in *C. surera* in a freshwater environment. Finally, it is tempting to suggest for future studies that sulfated polysaccharides could have, in this case, unknown masked functions, not related to salinity adaptation.

## Author Contributions

PA, JA, and ME performed the experiments, VC analyzed and discussed the phylogenetic data, MC programmed the experiments, JE and MC dicussed the results and wrote the manuscript.

## Conflict of Interest Statement

The authors declare that the research was conducted in the absence of any commercial or financial relationships that could be construed as a potential conflict of interest. The reviewer KM and handling Editor declared their shared affiliation.

## Supplementary Material

FIGURE S1 Maps showing the collecting site of the green macroalgae *Cladophora surera* located in Buenos Aires Province, Argentina **(A)**. Specimens of a green macroalgae were collected in a fresh-water environment of Quequén Grande river (38°27′39′′ S 58°45′39′′ W) **(B)**.
