# Supplementary figures and images for: Sulfated Polysaccharides in the Freshwater Green Macroalga Cladophora surera Not Linked to Salinity Adaptation
Source: Front Plant Sci. 2017 Nov 13;8:1927. doi: 10.3389/fpls.2017.01927 (PMC5694217; doi:10.3389/fpls.2017.01927)

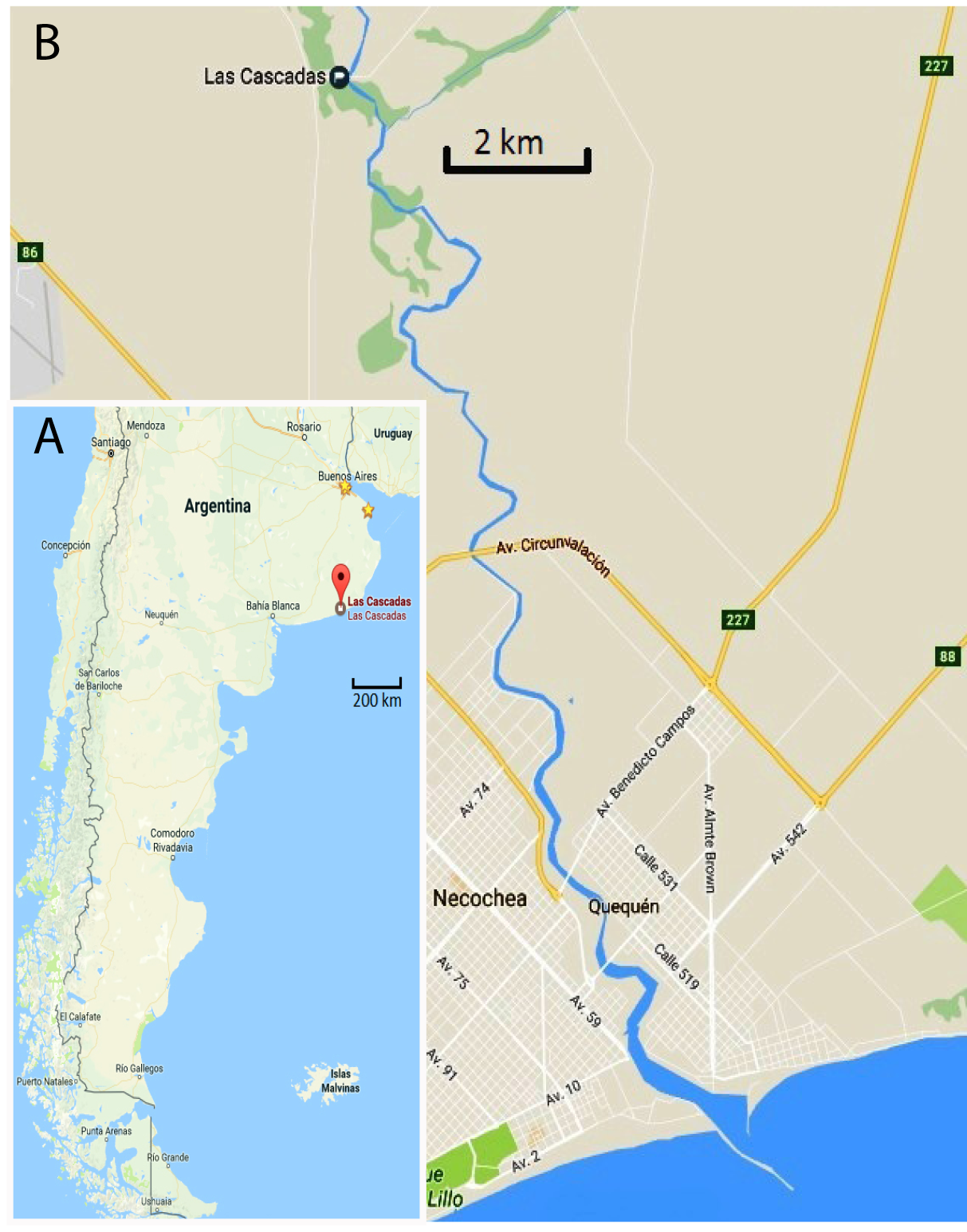

Supplement: FIGURE S1 — Maps showing the collecting site of the green macroalgae Cladophora surera located in Buenos Aires Province, Argentina (A). Specimens of a green macroalgae were collected in a fresh-water environment of Quequén Grande river (38°27′39′′ S 58°45′39′′ W) (B). [file Image_1.tif]

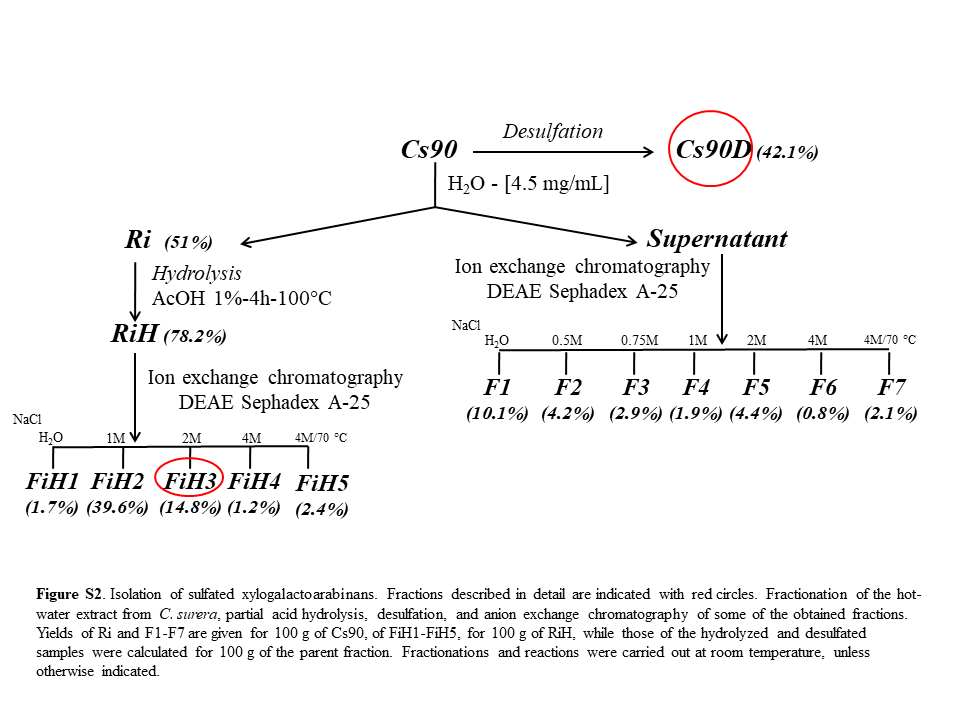

Supplement: Supplementary file 2 [file Image_2.tif]
